# Process parameters impacting product quality

**DOI:** 10.1186/1753-6561-9-S9-O7

**Published:** 2015-12-14

**Authors:** Jan Bechmann, Frederik Rudolph, Lucy Gebert, Jochen Schaub, Benedikt Greulich, Michael Dieterle, Harald Bradl

**Affiliations:** 1 Boehringer Ingelheim Pharma GmbH & Co. KG, Biberach

## Background and novelty

Product quality is a result of the entire production process including protein sequence, cell substrate and process parameters. Many of the desired product properties are defined by posttranslational modifications with impact on biological activity, immunogenicity, half-life or stability. In-depth process understanding enables the targeted modulation of product quality attributes by rationally designed bioprocesses. This is valuable for new biological molecules in order to improve efficacy, reduce side effects, access new patient populations. For biosimilars this allows developing into defined quality attribute profiles. The identification of suitable process parameters and media compositions to modulate quality attributes is challenging due to the complexity of cell culture processes.

Here, this challenge was approached by comprehensive data analysis, in-depth characterization of charge variant formation and high-throughput screening of process parameters and media compounds.

## Experimental approach

The impact of process parameters on product quality attributes was analyzed with special focus on acidic charge variants and glycosylation pattern. Initially a database was created including process and analytical data from twelve projects. Data sets of more than 2500 fed-batch processes with 6300 analytical data sets enabled a cross-project analysis and correlation of process parameters with product quality attributes.

The formation of charge variants was explored by uni- and multivariate techniques within the database to identify potentially impacting process parameters. These were then further investigated in experimental work. Cell culture parameters impacting growth and product formation rates like media osmolality and pH profiles were tested in bioreactor cultivations. In addition, post-harvest experiments exploring different pH, temperature, light and buffer conditions were studied in storage stress studies. Data from both studies were integrated to establish predictive modeling of charge variant formation in upstream process supernatants.

In addition, the impact of cell culture conditions and media compounds on the glycosylation pattern was assessed by an integrated screening approach. Multi parallel small scale bioreactors, robotics based product capture and high throughput analytics were combined to minimize hands-on-time to gain data for correlation analysis.

## Results and discussion

Said setups supported the identification of numerous media supplements and upstream process conditions that were applied for rational modulation of glycosylation patterns and charge variants. For the latter, the analysis of formation kinetics enabled modeling of charge variant formation in process supernatants. A mechanistic model was established based on first order degradation kinetics. Data from post-harvest experiments was used to analyze dependencies of the molecule specific degradation rate (qn) on pH and temperature (T). Here, an exponential modulation by both process variables was found. The degradation rate q is further modulated by cell culture osmolality in a linear manner (compare equation 1). With the resulting model the prediction of final acidic molecule concentrations for five different IgG molecules would be predicted with a RMSEP = 2.5 % based the process variables product concentration, viable cell density, pH, temperature, bioreactor volume and osmolality in supernatants. The model clearly demonstrated that the largest impact on the final abundance of acidic molecule variants is product formation kinetics. Constant protein synthesis supplies fresh monoclonal antibody to the cell culture supernatant and thus dilutes the continuously formed acidic molecule variants. It was shown that process formats that modulate growth rates can especially facilitate this.

(1) $$\text{q}={\text{q}}_{\text{n}}\cdot 5.4\cdot 10^{-5}\cdot \text{e}\left(0.109\text{T}+0.811\text{pH}\right)\cdot \left(2,25-0,0035\cdot \text{Osmo}\right)$$

**Figure 1:**
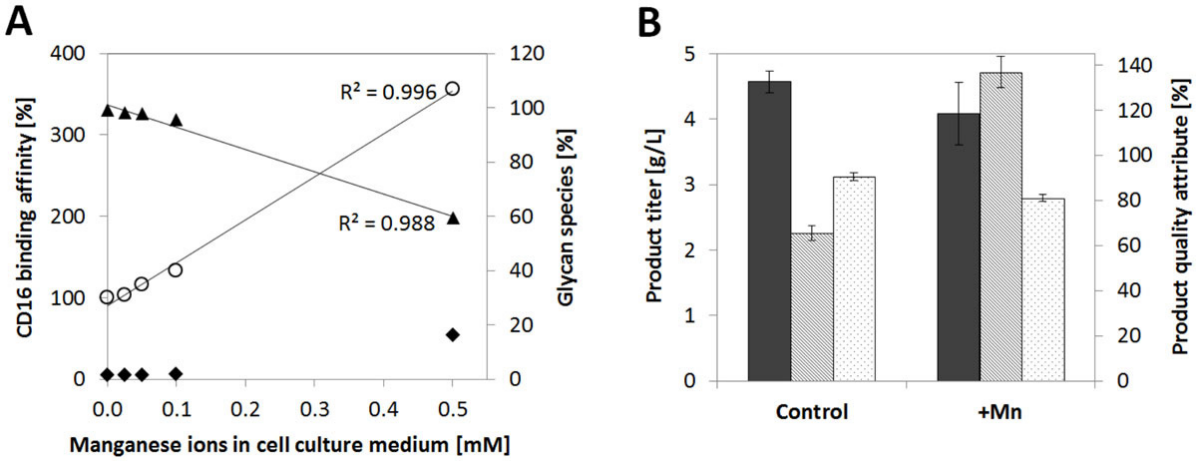
**Impact of manganese concentration in cell culture media on fucosylation, mannosylation and CD16 binding affinity of produced monoclonal antibodies**. A linear dependency of CD16 binding affinity (empty circles) and fucosylation (filled triangles) with manganese levels was characterized in 14-day fed-batch experiments in 15 mL-scale bioreactors (a). Mannosylation (filled diamonds) did not correlate with manganese concentrations in a strictly linear manner. Results from the screening experiment were verified with a different cell line in 2 L- scale (b). Final harvest titers (filled bars) were not impacted by manganese additions, while CD16 binding affinity of the product could be doubled (striped bars) by reducing fucosylation levels (dotted bars) by 10 % with 150 µM manganese as media supplement. Error bars indicate the standard deviation of biological duplicates.

Moreover, a case study focusing on the optimization of glycan patterns and antibody dependent cellular cytotoxicity by using metal ions as media supplements was presented. Data provided by Gramer et al. (2011) clearly indicated the linear correlation of manganese levels in cell culture media with non-fucosylated N-glycan species within a range from 0 to 40 µM. The described effect could be demonstrated up to 500µM manganese resulting in a reduction of fucosylation from 95 % to 60 %in 15 mL scale fed-batch cultivations. In contrast, high mannose type glycans were increased to only 15 %. By this increase in the non-fucosylated glycan fraction, CD16 binding affinity of the product molecule could be increased up to 350 % (the linear correlation of CD16 binding affinity and defucosylated N-glycan levels is described in literature, e.g. by Chung et al. (2012)) by increased manganese concentrations in cell culture media. Results obtained in 15 mL-scale were then verified in a 2 L-scale fed-batch processes. Cell lines expressing a different recombinant IgG molecule were grown in a cell culture medium containing 150 µM manganese. As shown in Figure 1, this media tool successfully enabled doubling the CD16 binding affinity of the molecule caused by a reduction of fucosylation by 10 %.

The overall derived database and toolbox is applied for ongoing projects for fine tuning of product quality attributes to meet desired characteristics. After gap analysis, process parameters can be chosen for application in process development to finally achieve high quality products.

## References

[B1] Chung S Quarmby V Gao X Ying Y Lin L Reed C Fong C Lau W Qiu ZJ Shen A Vanderlaan M Song A Quantitative evaluation of fucose reducing effects in a humanized antibody on Fcγ receptor binding and antibody-dependent cell-mediated cytotoxicity activities MAbs 2012 4 326 340 22531441 10.4161/mabs.19941 PMC3355491

[B2] Gramer MJ Eckblad JJ Donahue R Brown J Shultz C Vickerman K Priem P van den Bremer ETJ Gerritsen J van Berkel PHC Modulation of antibody galactosylation through feeding of uridine, manganese chloride, and galactose Biotechnol Bioeng 2011 108 1591 1602 21328321 10.1002/bit.23075

